# Rule-Based Knowledge Acquisition Method for Promoter Prediction in Human and *Drosophila* Species

**DOI:** 10.1155/2014/327306

**Published:** 2014-01-29

**Authors:** Wen-Lin Huang, Chun-Wei Tung, Chyn Liaw, Hui-Ling Huang, Shinn-Ying Ho

**Affiliations:** ^1^Department of Management Information System, Asia Pacific Institute of Creativity, Miaoli 351, Taiwan; ^2^School of Pharmacy, College of Pharmacy, Kaohsiung Medical University, Kaohsiung 807, Taiwan; ^3^Institute of Bioinformatics and Systems Biology, National Chiao Tung University, Hsinchu 300, Taiwan; ^4^Department of Biological Science and Technology, National Chiao Tung University, Hsinchu 300, Taiwan

## Abstract

The rapid and reliable identification of promoter regions is important when the number of genomes to be sequenced is increasing very speedily. Various methods have been developed but few methods investigate the effectiveness of sequence-based features in promoter prediction. This study proposes a knowledge acquisition method (named PromHD) based on if-then rules for promoter prediction in human and *Drosophila* species. PromHD utilizes an effective feature-mining algorithm and a reference feature set of 167 DNA sequence descriptors (DNASDs), comprising three descriptors of physicochemical properties (absorption maxima, molecular weight, and molar absorption coefficient), 128 top-ranked descriptors of 4-mer motifs, and 36 global sequence descriptors. PromHD identifies two feature subsets with 99 and 74 DNASDs and yields test accuracies of 96.4% and 97.5% in human and *Drosophila* species, respectively. Based on the 99- and 74-dimensional feature vectors, PromHD generates several if-then rules by using the decision tree mechanism for promoter prediction. The top-ranked informative rules with high certainty grades reveal that the global sequence descriptor, the length of nucleotide A at the first position of the sequence, and two physicochemical properties, absorption maxima and molecular weight, are effective in distinguishing promoters from non-promoters in human and *Drosophila* species, respectively.

## 1. Introduction

**Figure 1:**
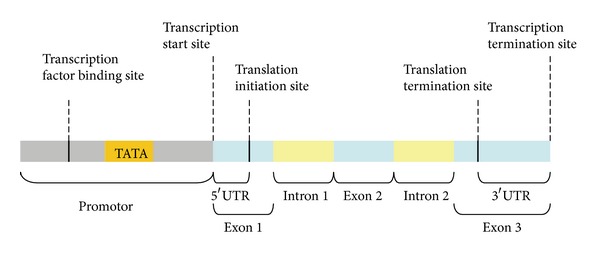
The promoter of a DNA sequence containing a transcription factor binding site and a TATA box is immediately upstream to a transcription start site.

Gene expression is often regulated by the transcription rate, which is largely controlled by the binding of RNA polymerase II (Pol II) to the regulatory regions of DNA sequences in eukaryotic cells [1]. The regulatory regions (called promoters) that contain a transcription factor binding site and a TATA box are immediately upstream of transcription start sites at which transcription factors and Pol II are accumulated to initiate the transcription (Figure 1) [2, 3]. Promoters are extremely diverse and difficult to identify experimentally using specific sequence patterns or motifs [3, 4]. Therefore, the identification of promoters is very challenging, especially in the sequencing of eukaryotic genomes. Some methods for predicting promoters have been developed, and these methods may be categorized into the following four classes according their types of sequence features (see Table 1).

1. *Context-Feature Class.* Context features are contents of the documents that are represented by basic unit DNA words called *k*-mer motifs (*k*-base-long nucleotide sequences) [5]. Besides *k*-mer frequency, some features based on *k*-mer motifs are also used in promoter prediction including transition [6], distribution [6], entropy density profile (EPD) [6], codon-position-independent frequencies of mononucleotides [6, 7], digitized DNA sequence [8], position-specific propensity [9, 10], relative entropy [10], and flanking genomic sequence [4].
2. *Signal-Feature Class.* Powerful biological signals contain core-promoter elements [11], some short modular transcription factor binding sites [12], and CpG islands [13, 14]. The core-promoter elements that play important roles in the assembly of transcriptional machinery contain the TATA box, the exons region [15, 16], the intron region (initiator sequences), [15, 17–19], downstream promoter elements [20], a TFIIB recognition element [11], motif ten element [21], and CCAAT box [19, 22, 23]. The TATA box, initiator sequences, and consensus sequences for transcription factor binding sites are often used in various promoter recognition methods [24]. However, these features have been confirmed to exist only in a small proportion of all human promoters [25, 26].
3. *Structure-Feature Class.* Many physical and structural properties of DNA sequences are estimated. They include DNA curvature [27], flexibility [22, 23], denaturation values [28, 29], base stacking energy [16, 28], stabilizing energy of Z-DNA [30], Z-DNA [31], and radical cleavage intensity [28, 32]. In particular, McPromoter [29] is a probabilistic promoter predictor that uses a neural network to combine the sequence features and structural profiles, such as those of DNA bend ability or GC structure, in promoter prediction.
4. *Epigenetic-Feature Class.* Few promoter prediction methods utilize epigenetic information [22, 33]. For example, HMM-SA is a supervised learning method for predicting promoters and enhancers from their unique chromatin modification signatures [33]. Similarly, CoreBoost_HM systematically analyzes different chromatin features for promoter prediction [22].

Table 2 lists some representative methods that use the above four types of features in combination with effective classifiers to predict promoters. These classifiers involve Fisher's linear discriminant algorithm [6], the hidden Markov model [22, 23], the AdaBoost algorithm [8], decision trees [18], relevance vector machines [34], the expectation maximization algorithm [35, 36], artificial neural networks [12, 15, 17, 19, 29, 37], support vector machines (SVM) [38, 39], artificial immune recognition systems [40], and others. Recently, the use of ensemble classifiers has become popular in promoter prediction systems. For example, CoreBoost [23] and CoreBoost_HM [22] used ensemble classifiers to improve prediction performance. These methods apply boosting techniques with stumps to extract sequence features, including the core-promoter elements score, transcription factor binding site density, the DNA flexibility of promoter sequences, Markovian log-likelihood ratio scores, *k*-mer frequencies, and epigenetic features.

Even though many promoter prediction methods have been developed, the effectiveness of used features in identifying promoters still needs to be explored. However, accurate promoter prediction relies largely on feature extraction and model selection [3]. Currently, Prom-Machine [39] simply selected 128 four-mer motifs and then utilized these motifs in conjunction with SVM to improve prediction sensitivity and specificity toward the DNA sequences of the five following species: plants, *Drosophila*, human, mouse, and rat. Additionally, one recently published method, FSPP [41], used both filter and wrapper algorithms to select effective feature subsets from 13 kinds of structural features, including DNA-bending stiffness, duplex free energy, and duplex disrupt energy to improve further the sensitivity and accuracy of promoter prediction. Our previous method, ProPolyII [42], selects a small number of sequence-based features to improve prediction performance in human species.

These methods motivate this work to focus on feature selection and effectiveness evaluation of the selected features in promoter prediction. This work presents a knowledge acquisition method (named PromHD) based on if-then rules for promoter prediction in human and *Drosophila* species. The knowledge can be revealed from three aspects: (1) identified informative DNA sequence descriptors (DNASDs), (2) rules of distinguishing promoter from nonpromoter, and (3) further analysis of distinguishable mechanism using DNASDs. PromHD utilizes a reference feature set of 167 DNASDs, comprising three descriptors of physicochemical properties (absorption maxima, molecular weight, and molar absorption coefficient) [43, 44] with 128 top-ranked frequency descriptors of 4-mer motifs and 36 global sequence descriptors. To the best of our knowledge, these three descriptors of physicochemical properties are used herein for the first time in identifying promoter DNA sequences and their sequence-based representation differs from the structural profiles of McPromoter [29]. The 128 top-ranked frequency descriptors of 4-mer motifs are extracted from 256 4-mer combinations of nucleotides (4-base-long nucleotide sequences) according to the scores that equal the difference between the occurrence frequencies of the 4-mer motif in the positive and negative datasets [45, 46]. PromHD further utilizes an effective feature mining algorithm (called DNASDmining), which is based on an inheritable biobjective genetic algorithm [47, 48], to mine informative DNASDs.

A total of 1871 human and 1926 *Drosophila* promoter sequences were downloaded from the Eukaryotic Promoter Database [49], which is a database containing over 4800 promoters from various species. The same numbers of nonpromoters in human and *Drosophila* species were collected to evaluate the proposed PromHD method. Accordingly, PromHD identifies two subsets of 99 and 74 DNASDs and yields test accuracies of 96.4% and 97.5% in human and *Drosophila* species, respectively, which are better than those of SVM-4mer (91.0% and 94.6%) and SVM-GSD (93.6% and 89.2%), respectively. Based on each of the 99- and 74-dimensional feature vector, PromHD uses the decision tree method C5.0 [50] to generate several if-then rules. The top-ranked rules reveal that the global sequence descriptor, the length of nucleotide A at the first position of the sequence, is efficient in distinguishing human promoters from nonpromoters, consistent with the findings of Wang et al. and Zhao et al. [22, 23]. Alternatively, the top-ranked rules in *Drosophila* species reveal that two physicochemical properties, absorption maxima and molecular weight, are effective in distinguishing promoters from nonpromoters. Further analysis of the two feature subsets shows that 32 features are common including three physicochemical properties, 14 descriptors of 4-mer motifs, and 15 global sequence descriptors. When the three descriptors of physicochemical properties are excluded, PromHD with the remaining 96( = 99 − 3) and 71( = 74 − 3) DNASD features yield test accuracies of 94.4% and 95.5% in human and *Drosophila* test datasets, respectively. The prediction accuracies fall by 2.0% ( = 96.4% − 94.4% and = 97.5% − 95.5%), reconfirming the three physicochemical properties are obviously effective in distinguishing promoters from nonpromoters in human and *Drosophila* species. The promoter prediction system by using the PromHD method has been implemented at http://iclab.life.nctu.edu.tw/promhd.

## 2. Materials and Methods

**Figure 2:**
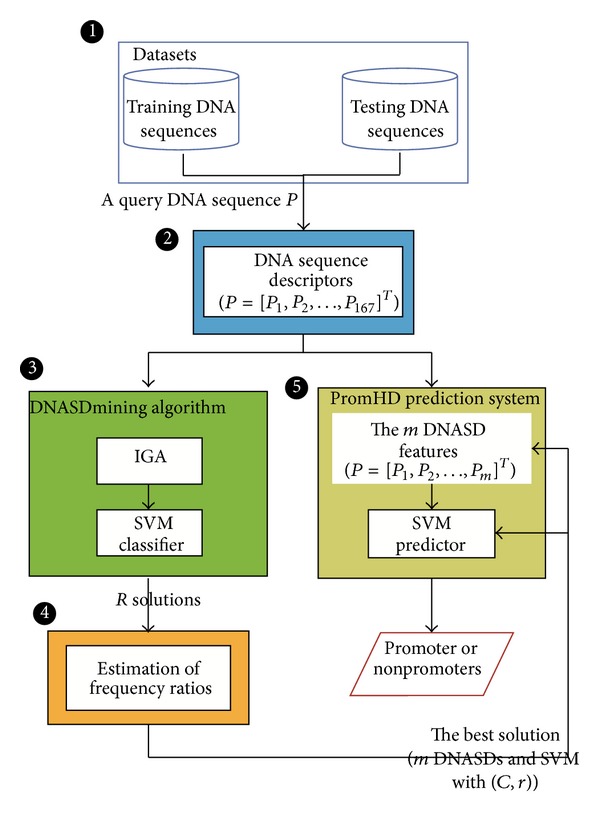
A block diagram of the PromHD method. The block diagram mainly contains the following important parts: (1) datasets, (2) DNA sequence descriptors, (3) DNASDmining algorithm, (4) estimating appearance-frequency ratios, and (5) PromHD prediction system.

In this work, a block diagram is used to illustrate the main components of the proposed PromHD method.  Figure 2 presents five main components, which are datasets, DNA sequence descriptors, DNASDmining algorithm, estimating appearance-frequency ratios, and the PromHD prediction system.

### 2.1. Datasets

More than 4800 eukaryotic Pol II promoters from many species have been collected in the Eukaryotic Promoter Database (http://epd.vital-it.ch) in May 2013 [49], in which the transcription start site was determined experimentally and the numbers of promoters in the human and *Drosophila* species greatly exceed those in other species. Therefore, two datasets, HP (1871 human promoters and 1871 nonpromoters), and DP (1926 *Drosophila* promoters and 1926 nonpromoters), are established and used in this work to evaluate the proposed PromHD method. Segments of promoter sequences from −200 to +51 relative to a transcription start site [39] are collected. The nonpromoter sequences are extracted from the EMBL CDS (coding sequences) database (ftp://ftp.ebi.ac.uk/pub/databases/embl/cds/), which is a database of nucleotide coding sequences.

Both of the HP and DP datasets are equally divided into two subsets—one for training (learning) (HPL and DPL) and the other for independent testing (HPT and DPT). The learning dataset is done with the purpose of identifying a small set of DNASDs and finding the best parameters of a SVM to train the complete dataset [57, 58] (see Evaluation Measures). The sequences in the training and test datasets are randomly and near-equally partitioned. The numbers of DNA sequences within promoter and nonpromoter classes are presented in Supplementary Table S1 available online at http://dx.doi.org/10.1155/2014/327306.

### 2.2. DNA Sequence Descriptors

This work presents a reference feature set of 167 DNA sequence descriptors (DNASDs in Supplementary Material) that comprises three sequence descriptors of physicochemical properties, 128 top-ranked frequency descriptors of 4-mer motifs, and 36 global sequence descriptors. Therefore, a DNA sequence is represented as a 167-dimensional feature vector *P* = [*P*_1_, *P*_2_,…,*P*_*n*_]^*T*^, where *n* = 167. All of the features of P are rescaled into the range [0, 1] and are employed to SVM (Figure 2). The following three sections describe three subsets of DNASDs with using the sequence CATAGCCATTGCATGACCCG of length 20 as an example (called S20).

#### 2.2.1. Physicochemical Properties of Nucleotides

The physicochemical properties of the DNA structure of eukaryotic genomes are critical to promoter recognition. This study proposes a sequence-based set of three physicochemical properties of nucleotides (http://www.geneinfinity.org) for designing prediction features that are used to distinguish promoters from nonpromoters. The three DNA sequence descriptors, denoted as *D*_AM_, *D*_MW_, and *D*_MAC_, are derived from the three physicochemical properties—absorption maxima, molecular weight, and molar absorption coefficient, respectively—by averaging over a nucleotide sequence [59]. The three descriptors are the attributes of the subvector [*P*_1_, *P*_2_, *P*_3_] in the reference set of 167 DNASDs.

The sequence S20 has five As, seven Cs, four Gs, and four Ts. With reference to Table 3, the values of the absorption maxima (determined at pH 7.0) for nucleotides A, C, G, and T are 259, 271, 253, and 267, respectively. Accordingly, the descriptor *D*_AM_ has a value 263.6 = (5 × 259 + 7 × 271 + 4 × 253 + 4 × 267)/20. The other two descriptors *D*_MW_ and *D*_MAC_ have values 484.95 and 11950, respectively.

#### 2.2.2. Global Sequence Descriptors

The global description of promoter/nonpromoter sequences contains four parts, entropy density profile (EDP), composition, transition, and distribution of DNA nucleotides [6]. The EDP model is a global statistical description for a DNA sequence, based on Shannon's artificial linguistic description for a DNA sequence of finite length [60]. Let *q*_*i*_ be the frequencies of occurrence of nucleotides in a promoter/nonpromoter sequence, where *i* is the index that specifies the nucleotides (A, C, G, T). Six EDPs, *D*_EH_, *D*_EQ_, *D*_EA_, *D*_EC_, *D*_EG_, and *D*_ET_, correspond to the six attributes of the subvector [*P*_4_,…, *P*_9_], defined as follows:

(1) DEQ=qA2+qC2+qG2+qT2,  DEH=−∑iqilog⁡⁡qi,DEi=−1DEHqilog⁡⁡qi,

where *D*_EH_ is the Shannon entropy and *D*_EQ_ is a statistical quantity.

The composition is used to measure the frequency of occurrence of each kind of letters in the sequences, and thus herein it is the *q*_*i*_ in (1). Additionally, the four frequencies *q*_*i*_ are also called 1-mer motifs of the nucleotides (A, T, C, and G), denoted as *D*_C1_ (A), *D*_C1_ (T), *D*_C1_ (C), and *D*_C1_ (G), and correspond to the four attributes of [*P*_10_,…, *P*_13_].

The third part, transition *T*(*α*, *β*), characterizes the percent frequency with which *α* is followed by *β* or *β* is followed by *α*. The six transition frequencies, *D*_*T*_(A, C), *D*_*T*_(A, G), *D*_*T*_(A, T),*D*_*T*_(C, G), *D*_*T*_(C, T), and *D*_*T*_(G, T), correspond to the six attributes of [*P*_14_, …, *P*_19_]. For example, for the S20 sequence, there are four transitions of this type *T*(A, C), **CA**TAGC**CA**TTG**CA**TG**AC**CCG in bold style; that is, the value of *D*_*T*_(A, C) is 21.0526 ( = 4/19) × 100.00.

The fourth part of the global description, distribution, measures the chain length within which the first, 25%, 50%, 75%, and 100% of certain type of letters are located, respectively. For example, for the S20 sequence, the first, 25%, 50%, 75%, and 100% of the nucleotide A are located within the second, 4th, 8th, 13th, and 16th nucleotides, respectively. So, the five distributions of the nucleotide A, *D*_*D*_(A, 1st), *D*_*D*_(A, 25%), *D*_*D*_(A, 50%), *D*_*D*_(A, 75%), and *D*_*D*_(A, 100%), have values of 10 ( = 2/20∗100), 20 ( = 4/20∗100), 40 ( = 8/20∗100), 65 ( = 13/20∗100), and 80 ( = 16/20∗100), respectively. A total of 20( = 4 × 5) distributions corresponding to [*P*_20_,…, *P*_39_] when four types of nucleotides are considered.

#### 2.2.3. Frequency Descriptors of 4-Mer Motifs

The number of 4-mer combinations of nucleotides (4-base-long nucleotide sequences) is 256. Prom-Machine [39] uses top 128 of the 256 4-mer motifs to improve prediction sensitivity and specificity. Our earlier works estimated the scores of amino acids [45] and of GO terms [46] for predicting DNA-binding proteins and nonclassical secretory proteins, respectively. That motives this work, in which a score for each 4-mer motif is calculated and the 128 top-ranked 4-mer motifs based on those scores are identified. The score is the difference between the occurrence frequencies. A detailed description follows.

**Step 1**

The occurrence frequencies *f*_*ω*_ and *F*_*ω*_ are those of the *ω*th 4-mer motif in all training promoter (positive) and nonpromoter (negative) sequences, respectively, where *ω* = 1, 2, …, 256. For example, TATA is the 199th 4-mer motif, that is, *ω* = 199, and its occurrence frequencies in the positive and negative classes of the HPL dataset are *f*_199_ = 84 and *F*_199_ = 453, respectively (see Table 4).

**Step 2**

Calculate the total numbers of occurrences of 256 4-mer motifs in the positive and negative classes, Σ*f*_*ω*_ and Σ*F*_*ω*_. For example, the total numbers of occurrences of 256 4-mer motifs in the positive and negative classes of the HPL dataset are 29104 and 137017, respectively.

**Step 3**

The two proportional frequencies of occurrence in the positive and negative classes for each 4-mer motif are the values of *f*_*ω*_/Σ*f*_*ω*_ and *F*_*ω*_/Σ*F*_*ω*_, respectively. For example, the proportional frequencies of occurrence of TATA are 0.002892 ( = 84/29104) and 0.003309 ( = 453/137017) in the positive and negative classes, respectively.

**Step 4**

The score for each 4-mer motif is the absolute value |·| of the difference between the proportional frequencies of occurrence in the positive class and that in the negative class. For example, the score of TATA is 0.000417 ( = |0.00309 − 0.002892|).

**Step 5**

Normalize scores of all 256 4-mer motif into the range [0, 13000], and represent them as {*θ*_1_, *θ*_2_,…, *θ*_*n*_}. The normalized score is also called the frequency descriptor of the 4-mer. For instance, the score of TATA motif is 111 (see Table 4).

**Step 6**

All 256 frequency descriptors are ranked in descending order. The top 128 motifs with descriptors are denoted as *D*_C4_(·) and they correspond to [*P*_40_, …, *P*_167_]. For example, the descriptor of the well-known TATA box, as shown in Table 4, is *D*_C4_(TATA) = 111 and corresponds to *P*_137_ for the DPL dataset.

### 2.3. Proposed DNASDmining Algorithm

An efficient feature-mining algorithm, DNASDmining, for identifying a set of informative DNASDs is developed. The DNASDmining algorithm is an expansive version of an inheritable biobjective genetic algorithm, which is based on an intelligent genetic algorithm (called IGA) [47, 61], to identify a small number *m* out of *n* = 167 DNASDs. The feature selection is a combinatorial optimization problem Comb(*n*, *m*) with a huge search space of size Comb(*n*, *m*) = *n*!/(*m*!(*n* − *m*)!). The IGA, based on an orthogonal experimental design using a divide-and-conquer strategy and systematic reasoning, can efficiently solve the large combinatorial optimization problem to obtain the solution *S*_*r*_ to Comb(*n*, *r*). The mechanism can efficiently search for the next solution *S*_*r*+1_ to Comb(*n*, *r* + 1) by inheriting the last solution *S*_*r*_. DNASDmining obtains all solutions *S*_*r*_ from *r* = *r*_start_ to *r*_end_ one by one using IGA with the inheritable mechanism [47, 61].

#### 2.3.1. Feature Selection

The input of the DNASDmining algorithm is a training set of DNA sequences that are categorized into two classes—promoter and nonpromoter sequences. The output comprises a set of *m* informative DNASDs and the parameter settings (*C*, *γ*) of an SVM classifier. The SVM is a binary classifier of LIBSVM with a radial basis kernel function [62], where a kernel parameter and a cost parameter *C* are tuned by IGA. In this study, *γ* ∈ {2^−7^, 2^−6^,…, 2^8^} and *C* ∈ {2^−7^, 2^−6^,…, 2^8^}. The IGA-chromosome *S* comprises *n* binary IGA-genes *g*_*i*_ for selecting informative features and two 4-bit IGA-genes for encoding *γ* and *C*, where *i* = 1, 2,…, 167. The *i*th DNASD feature *P*_*i*_ is used in the SVM classifier if *g*_*i*_ = 1; otherwise, *P*_*i*_ is excluded (*g*_*i*_ = 0).  Figure 2 shows the sequence representation and the IGA-chromosome encoding method. Supplementary Table S2 lists the parameter settings of IGA, such as population size *N*_pop_ = 20. In this algorithm DNASDmining, *r*_start_ = 30, *r*_end_ = 100, and *G*_max⁡_ = 60 based on former experience.

**Step 1 (initiation)**

Randomly generate an initial population of *N*_pop_ individuals. All the *n* binary genes in the individual *S* have *r* 1's and *n* − *r* 0's where *r* = *r*_start_ and gen = 0.

**Step 2 (evaluation)**

Evaluate the fitness values fitness(*S*) of all individuals. The fitness function of this training model is the prediction accuracy of 10-fold cross-validation (see Evaluation Measures) using the SVM classifier with the *m* DNASDs, *γ*, and *C* by decoding the IGA-chromosome.

**Step 3 (selection)**

Use the simple ranking selection that replaces the worst *p*_*s*_ · *N*_pop_ individuals by the best *p*_*s*_ · *N*_pop_ individuals to form a new population where *p*_*s*_ is the selection probability.

**Step 4 (crossover)**

Select *p*_*c*_ · *N*_pop_ parents from the mating pool to perform orthogonal array crossover [47, 61] on the selected pairs of parents where *p*_*c*_ is the crossover probability.

**Step 5 (mutation)**

Apply the swap mutation operator to the randomly selected *p*_*m*_ · *N*_pop_ individuals in the new population where *p*_*m*_ is the mutation probability. To prevent a decline in the best fitness value, mutation is not applied to the best individual.

**Step 6 (termination test)**

If *gen* = *G*_max⁡_, then output the best individual as *S*_*r*_. Otherwise, increase the number gen by one, and go to Step 2.

**Step 7 (inheritance)**

If *r* < *r*_end_, then randomly change one bit in the binary genes of each individual from 0 to 1; increase the number *r* by one and let gen = 0, and go to Step 2.

**Step 8 (decoding chromosome)**

Let *S*_*m*_ be the most accurate solution with *m* selected DNASDs among all solutions *S*_*r*_ Obtain the *m* informative features and values of the parameters *γ* and *C*.

**Step 9 (system uncertainty)**

Perform Steps 1–8 for *R* independent runs to obtain the best solution, *S*_*m*_, and the associated parameter settings of the SVM classifier (see Section 2.4).

#### 2.3.2. Evaluation Measures

The independent dataset test, subsampling or *N*-fold (e.g., 5- or 10-fold) cross-validation test, and the jackknife test are often used to examine the accuracy of a statistical prediction method [63]. The jackknife test is deemed to be the least arbitrary method that can always yield a unique result for a given benchmark dataset [64]. The *N*-fold cross-validation test is used to estimate the error that is involved in the predictions and thus it is also used for model selection [65]. In this work, 10-fold cross-validation scheme is used. Additionally, the independent dataset test is also used in this work to avoid overestimating the success rate of the training model.

Overall accuracy (ACC), sensitivity (SN), and specificity (SP) are three quality measures that are widely used to evaluate the performance of promoter prediction methods [66]. This work also utilizes Matthews correlation coefficient (MCC) to measure the overall performance of the prediction models. It takes into account true and false positives and negatives and is generally regarded as a balanced measure which can be used even if the classes are of very different sizes [67]. These measures are defined as below:

(2) ACC=(TP+TN)(TP+FP+TN+FN),SN=TP(TP+FN),SP=TN(TN+FP),MCC   =(TP∗TN+FP∗FN)(TP+FP)∗(TP+FN)∗(TN+FP)∗(TN+FN).

TP, TN, FP, and FP stand for true positive, true negative, false positive, and false negative, respectively. The MCC returns a value in the range [−1, 1]. A value of 1 indicates a perfect prediction; 0 indicates a random prediction, and −1 indicates an inverse prediction.

### 2.4. Estimating the Appearance-Frequency Ratios

A total of *R* experimental runs are executed to obtain the best solution in this work due to the system uncertainty of the IGA-based feature selection algorithm. That means that *R* solutions are generated and each solution comprises a subset of *m*_*k*_ selected DNASDs for *k* = 1,…, *R*. The best solution must have both high prediction accuracy and a high appearance-frequency value. The estimation procedure is further described below.

**Step 1**

Calculate the appearance-frequency *Af*_*j*_ according to (3) for each of *j* = 1, 2,…, 167 DNASDs in all *R* runs. For example, *R* = 20 and the *D*_*MW*_ descriptor that is the 129th out of 167 DNASDs appears 19 times, so its appearance-frequency *Af*_129_ = 19.

**Step 2**

Sum all appearance frequencies to obtain *AF*( = Σ*f*_j_) according to (4).

**Step 3**

Calculate the appearance-frequency *Af* ^*k*^ for each run, *k* = 1,…, *R* using (5).

**Step 4**

**Figure 3:**
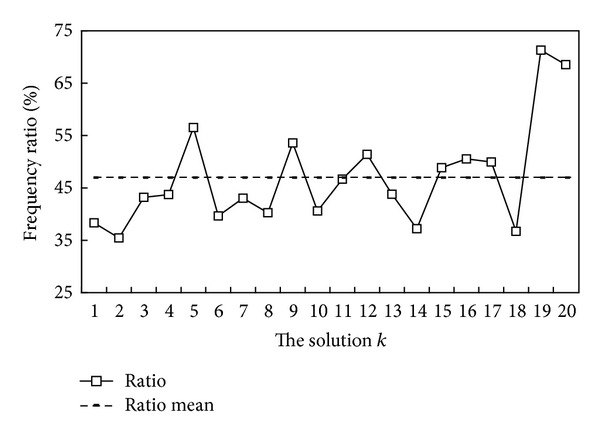
Appearance-frequency ratios of *R* DNASDmining solutions, where *k* = 1, 2,…, *R*. The mean frequency ratio is 47.0% for HPL dataset.

Calculate the appearance-frequency ratio *ℜ* ^*k*^ = *Af* ^*k*^/*AF* and the mean value *ℜ* ^*m*^. For instance, Figure 3 displays the mean *ℜ* ^*m*^ = 47.0% for the HPL dataset.

**Step 5**

Select the candidate solutions *S*_*k*_ from the *R* runs whose appearance-frequency ratios *ℜ* ^*k*^ are larger than the mean value *ℜ* ^*m*^. For instance, the appearance-frequency ratio *ℜ* ^*k*^ for *k* = 5, 9, 12, 15, 16, 17, 19, 20 exceeds the mean 47.0%, as shown by Figure 3. Thus, these eight solutions are selected as the candidate solutions.

**Step 6**

The best solution is the candidate solutions *S*_*k*_ with the highest prediction accuracy. For the above example, the 5th candidate solution *S*_5_ having the highest accuracy 98.9% is selected as the best solution of the DNASDmining algorithm, where (*C*, *γ*) = (2^7^, 2^−5^),

(3) $$\begin{array}{c} Af_{j}=\overset{R}{\underset{k=1}{\sum }}af_{jk},\;j=1,2,\ldots ,167, \end{array}$$

(4) $$\begin{array}{c} AF=\overset{167}{\underset{j=1}{\sum }}Af_{j}, \end{array}$$

(5) $$\begin{array}{c} Af^{k}=\overset{167}{\underset{j=1}{\sum }}af_{jk},\;k=1,2,\ldots ,R. \end{array}$$

### 2.5. PromHD Prediction System

The PromHD prediction system is implemented by using a SVM classifier with a subset of m DNASDs, where the parameter settings of SVM and the value of m are determined in the training phase.  Figure 2 illustrates the prediction flowchart of PromHD. The input to this prediction system is a query DNA sequence P. The output is the predicted classpromoter or nonpromoter. The prediction procedure is described as follows.

**Step 1**

The query DNA sequence is represented as a 167-dimensional DNASD feature vector **P** = [*P*_1_, *P*_2_,…,*P*_167_]^*T*^.

**Step 2**

The *m* informative DNASDs are selected from **P**, where *m* = 99 and 74 for human and *Drosophila* DNA sequences, respectively.

**Step 3**

The *m* selected features are input to the trained SVM to classify **P** as a promoter or non-promoter.

## 3. Results and Discussion

### 3.1. Effectiveness of Informative DNASDs

**Figure 4:**
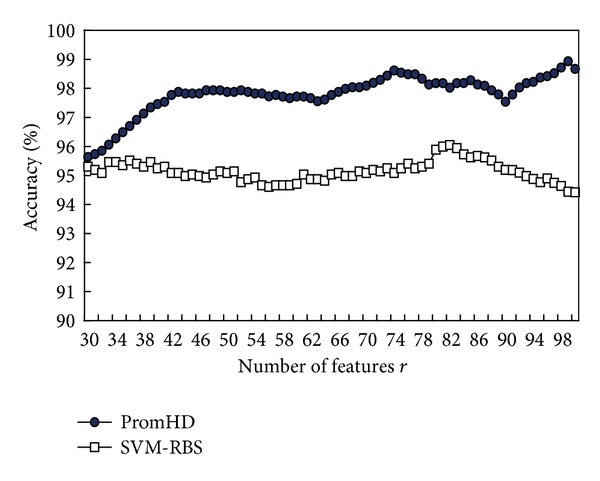
Training accuracies of the PromHD method and using SVM with a number *r* of selected informative features for the HPL dataset.

DNA sequences in this work are represented using 167-dimensional vectors of DNASDs. This work uses an efficient feature selection algorithm not only to select a subset of size *m* from the 167 DNASDs but also to design a SVM-based classifier simultaneously. To determine the candidate solution *S*_*r*_ in the DNASDmining algorithm, the prediction accuracy of 10-CV is used as a fitness function of the IGA.  Figure 4 shows the training accuracies of PromHD from *r* = 30 to 100 when applied to the HPL dataset and processed the 5th experimental run. These accuracies exceed those of SVM-RBS using SVM with a number *r* of selected informative DNASDs that are selected by the rank-based selection (RBS) method [68]. The RBS method is described below.

Each of the *n* = 167 DNASDs was ranked according to the accuracy of the SVM with the estimated single feature, where the best values of parameters (*C*, *γ*) were determined using a stepwise approach, where *γ* ∈ {2^−7^, 2^−6^,…, 2^8^}, and *C* ∈ {2^−7^, 2^−6^,…, 2^8^}. The 100 top-rank features *δ*_*i*_, *i* = 1,…, 100, were then picked, and the 30 top-ranked features with *r* = 30 were used as an initial feature set {*δ*_1_,…, *δ*_30_}. Consequently, the feature set with size *r* + 1 is incrementally created by adding the best feature *δ*_*r*+1_ (having the highest accuracy of SVM using 10-CV) from the remaining 100 − *r* features into the current feature set.

### 3.2. Comparison of Prediction Performance between PromHD and Other SVM-Based Methods

Two additional SVM-based classifiers, SVM-4mer and SVM-GSD, are applied for comparisons with SVM-RBS. The SVM-4mer and SVM-GSD methods are implemented by using the 128 top-ranked descriptors of 4-mer motifs and 36 global sequence descriptors, respectively, as input features to the SVM classifier without feature selection, respectively. The best values of parameters *C* and *γ* that are determined using a stepwise approach are used in the two SVM-based methods, where *γ* ∈ {2^−7^, 2^−6^,…, 2^8^} and *C* ∈ {2^−7^, 2^−6^,…, 2^8^}.

Tables 5 and 6 compare the three SVM-based methods in terms of performance when applied to the HP and DP datasets, respectively. SVM-GSD obtains the highest testing accuracy of 93.6% for the human species; SVM-4mer performs the best in *Drosophila* species. However, these testing accuracies are lower than those of PromHD, 98.9% and 96.4%, where *m* = 99 and 74 informative features are identified for human and *Drosophila* species, respectively. Additionally, the testing MCC values of PromHD are 0.927 and 0.949 for HPT and DPT, respectively, which exceed those of SVM-GSD (0.872 and 0.802), SVM-4-mer (0.823 and 0.830), and SVM-RBS (0.840 and 0.660), respectively. PromHD also yields high sensitivity (SN = 0.967 and 0.961) and specificity (SP = 0.960 and 0.988) performances when used with HPT and DPT datasets, respectively.

### 3.3. Rule-Based Knowledge

This work presents a knowledge acquisition method based on if-then rule for insight of promoter prediction mechanism. The knowledge can be revealed from three aspects: (1) identified informative DNASDs, (2) rules of distinguishing promoters from nonpromoters, and (3) further analysis of distinguishable mechanism using DNASDs. This rule-based knowledge acquisition method uses decision tree method C5.0 [50] to develop if-then rules of the 99- and 74-dimensional DNASD feature vectors in human and *Drosophila* species. Each if-then rule has two types, one for promoter (Ri-p) and the other for nonpromoter (Ri-n) prediction, where *i* is the rule number index. The selected DNASDs are *D*_*D*_(A, 1st) (the length of nucleotide A at the first position of the sequence), *D*_*D*_(C, 100%) (the length of nucleotide C at the last position of the sequence), *D*_C4_(GCTC) (the frequency descriptor of 4-mer GCTC), and *D*_MW_ (the physicochemical property of molecular weight) in human species.  Table 7 shows the interpretable rules as follows.

Rules in human species:

R1-p: if *D*_*D*_(A, 1st) > 0.0177542, then promoter prediction with CF = 0.928;
R2-n: if *D*_*D*_(A, 1st) ≤ 0.0177542, then nonpromoter prediction with CF = 0.999;
R3-n: if *D*_MW_ > 0.284657, *D*_*D*_(A, 1st) ≤ 0.0950018 and *D*_*D*_(C, 100%) ≤ 0.929016, then nonpromoter prediction with CF = 0.999;
R4-n: if *D*_C4_(GCTC) >0.0634629, *D*_MW_> 0.284657 and *D*_*D*_(A, 1st) 0.0950018, then nonpromoter prediction with CF = 0.974.

The CF is a certainty grade of this rule in the unit interval [0, 1]. The R1-p rule has a certainty grade of 0.928 to predict 935 ( = 50% × 1871) human promoters by using the *D*_*D*_(A, 1st) feature. With the same *D*_*D*_(A, 1st) feature, the second rule, R2-n, with a certainty grade of 0.999 can identify 864( = (96.2% − 50%) × 1871) nonpromoters. When applying these two rules, the rule-based classifier yields a prediction accuracy of 96.2%, reconfirming that the global sequence descriptor, the length of nucleotide A at the first position of the sequence, is an efficient feature in distinguishing human promoters from nonpromoters. When adding the third rule, PromHD further enhances the prediction accuracy up to 99.5%. For example, a query sequence P has normalized values of 0.0179, 0.9218, 0.2499, and 0.2823 for *D*_*D*_(A, 1st), *D*_*D*_(C, 100%), *D*_C4_(GCTC), and *D*_MW_, respectively. The classification procedure using the third rule R1-p (0.0179 > 0.0177542) predicts this query sequence to be a promoter.

Alternatively, the selected DNASDs are *D*_MW_ (the physicochemical property of molecular weight) and*D*_AM_ (the physicochemical property of absorption maxima) in *Drosophila* species. The interpretable rules, as shown in Table 7, are as follows.

Rules in *Drosophila* species:

R1-p: if *D*_MW_≤ 0.280113 and *D*_AM_ > 0.27604, then promoter prediction with CF = 0.997;
R2-n: if *D*_AM_≤ 0.27604, then nonpromoter prediction with CF = 0.999;
R3-n: if *D*_MW_> 0.280113, then nonpromoter prediction with CF = 0.997.

The rule-based classifier uses the first rule to predict 961 ( = 50% × 1922)  *Drosophila* promoters. The first two rules make the rule-based classifier have a prediction accuracy of 84.7% in *Drosophila* species. For example, a query sequence *P* has normalized values of *D*_MW_ and *D*_AM_, 0.4 and 0.3, respectively. The classification procedure using the third rule R3-n *D*_MW_( = 0.4)> 0.280113 predicts this query DNA sequence to be a nonpromoter.

### 3.4. Top 20 Descriptors of 4-Mer Motifs

Table 4 lists that the *D*_C4_(TGAA) and *D*_C1_(AAAG) descriptors have the maximum scores when applied to the HPL and DPL datasets, respectively. A comparison between the two sets of the top 20 descriptors of 4-mer motifs reveals two common 4-mer motifs. One is TGAA, which has scores of 1000 and 732; the other is TGAT, at ranks of 2 and 12 when used with HPL and DPL datasets, respectively. The descriptors of the well-known TATA motif are ranked at the 199th and 98th when applied for the HPL and DPL datasets, respectively.

The former descriptor *D*_*C*4_(TATA) ranking at the 199th is excluded out of the reference feature set due to the fact that only 128 top-ranked descriptors of 4-mer motifs are allowed to be included. This agrees closely with the findings of Gershenzon and Ioshikhes [11], who found that the TATA motif exists only in a small proportion of all human promoters. Additionally, only nine descriptors are included in each of the two feature subsets of *m* = 99 and 74 DNASD feature, which are marked with “+” Table 4. This main reason is that the DNASDmining feature selection algorithm considers a set of informative DNASDs at once, rather than individual DNASDs.

### 3.5. Analysis of the Identified DNASDs

The orthogonal experimental design with orthogonal array and factor analysis is an efficient method for simultaneously examining the individual effect of several factors on the evaluative function [47, 48]. In this study, the two levels of a factor represent its inclusion and exclusion of the feature in the feature selection using IGA [47, 48]. The factor analysis can quantify the effects of individual factors on the evaluation function, rank the most effective factors, and determine the best level for each factor for optimization of the evaluation function. The most effective factor has the largest main effect difference (MED) amongst the levels of a single factor.

**Figure 5:**
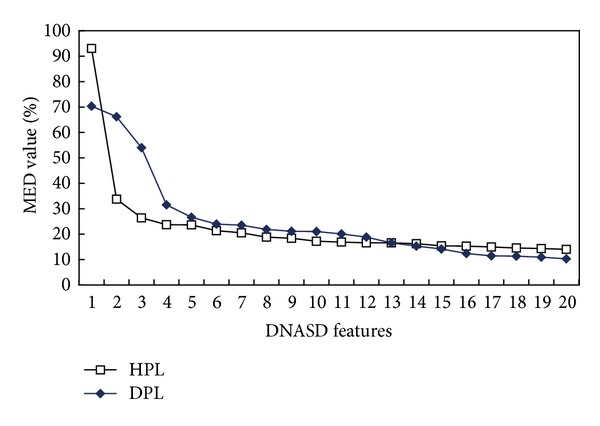
Top 20 DNASDs, which are ranked by MED values, for human and *Drosophila* training datasets. The MED values of the first two and four features exceed 30 when performing HPL and DPL datasets, respectively.

Figure 5 displays top 20 DNASDs when ranked in order of decreasing MED value. The MED values of the first two and four features exceed 30 when applied to the HPL and DPL datasets, respectively. The two features with the maximum MED values are *D*_*D*_(A, 1st) and *D*_*D*_(T, 1st), which are two distributions of the global description, respectively (Supplementary Table S3). Specifically, the *D*_*D*_(A, 1st) has the highest MED value of 93.1, meaning that the length of nucleotide A at the first position of the sequences can be used to distinguish promoters from nonpromoters in the human species. This result is consistent with the first if-then rule, R1-p (Table 7).

As for the four features with the MED values exceeding 30, they are the descriptors of the physicochemical properties (*D*_AM_, *D*_MW_, and *D*_MAC_) and *D*_C4_(AAGT), revealing that the three physicochemical properties of absorption maxima, molecular weight, and molar absorption coefficient can be used to distinguish promoters from nonpromoters in the *Drosophila* specie. The *D*_C4_(AAGT), a descriptor of the AAGT motif, has the fourth highest MED value of 31.5; however, it only has a score of 73 and a rank of 41, as shown in Table 4, revealing that DNASDmining can consider the internal correlation within relevant features rather than individual features using an efficient global optimization [45]. The transition descriptor *D*_*T*_(C, G) of the occurrence frequency of CG or GC has the fifth highest MED value of 26.6. This analytical result agrees with those obtained in other studies in [4, 19, 22, 23], which have found that GC content is effective in identifying promoter regions.

Supplementary Table S3 ranks all of the *m* = 99 informative DNASDs by MED value. They consist of three descriptors of physicochemical properties, four EDP descriptors, two composition descriptors, three transition descriptors, 14 distribution descriptors, and 73 4-mer frequency descriptors, denoted as 3(*D*_P_), 4(*D*_E_), 2(*D*_C1_), 3(*D*_*T*_), 14(*D*_*D*_), and 73(*D*_C4_), respectively, (see Table 8), where the abbreviations *D*_P_, *D*_C1_, *D*_C4_, *D*_E_, *D*_*D*_, and *D*_*T*_ represent the descriptors of the physicochemical property of nucleotides, the 1-mer motif, the 4-mer motif, EDP, distribution, and transition, respectively. On the other hand, the subset of *m* = 74 DNASDs comprises 3(*D*_P_), 2(*D*_E_), 2(*D*_C1_), 3(*D*_*T*_), 15(*D*_*D*_), and 49(*D*_C4_), shown in Table 7. Supplementary Table S4 ranks all of the 74 informative DNASDs by MED value.

### 3.6. Common DNASDs in Human and *Drosophila* Species

The percentages of common DNASDs in the two identified feature subsets are 32% ( = 32/99) and 43% ( = 32/74). The 32 common DNASDs, as shown in Table 8, comprise three descriptors of physicochemical properties, one EDP descriptor, two composition descriptors, one transition descriptor, 11 distribution descriptors, and 14 descriptors of 4-mer motifs, denoted as 3(*D*_P_), 1(*D*_E_), 2(*D*_C1_), 1(*D*_*T*_), 11(*D*_*D*_), and 14(*D*_C4_), respectively

All of the three descriptors of physicochemical properties, *D*_AM_, *D*_MW_, and *D*_MAC_, are ranked the first, second, and third for DPL and ranked the 5th, 6th, and 10th for HPL, respectively, consistent with the interpretation of if-then rules in Table 7. When the three descriptors of physicochemical properties are excluded, PromHD with the remaining 96( = 99 − 3) and 71( = 74 − 3) DNASD features yields test accuracies of 94.4% and 95.5% in human and *Drosophila* test datasets, respectively. The prediction accuracies fall by 2.0% ( = 96.4% − 94.4% and = 97.5% − 95.5%), reconfirming the three physicochemical properties are obviously effective in distinguishing promoters from nonpromoters in human and *Drosophila* species.

The one EDP descriptor *D*_EG_ is ranked the 13th and 32th for DPL and HPL, respectively, as shown in Supplementary Tables S3 and S4. The two compositions that are related to nucleotide A and G, denoted as *D*_C1_ (A) and *D*_C1_ (G), clearly contribute to promoter prediction, consistent with the findings of Wang et al. and Zhao et al. [22, 23]. The one transition descriptor *D*_*T*_(G, T) that characterizes the frequency of occurrence of two nucleotides GT or TG is ranked the 84th and 23th for human and *Drosophila* species, respectively. Among the 11 common distribution descriptors, *D*_*D*_(A, 1st), *D*_*D*_(T, 1st), and *D*_*D*_(G, 1st) are ranked the first, second, and third for the human species but the 46th, 20th, and 27th for *Drosophila* species, respectively. Two of the 14 common descriptors of 4-mer motifs, *D*_C4_(GAGC) and *D*_C4_(GAAG), not only have high scores of 592 and 699, respectively, but are also identified by PromHD to be informative DNASDs as can be seen by comparing Tables 4,  S3, and S4. Although the ranks of these common 32 DNASDs largely differ between human and *Drosophila* species, they form the six clusters of *D*_P_, *D*_E_, *D*_C1_, *D*_*T*_, *D*_*D*_, and *D*_C4_. The scenarios reveal that the six clusters are all useful for distinguishing promoters from nonpromoters in human and *Drosophila* species.

## 4. Conclusions

Promoter prediction is an important problem in elucidating the regulation of gene expression. Therefore, the development of a well-characterized promoter system is vital for synthetic biology applications. This proposed PromHD method presents a reference feature set of 167 DNASDs, utilizes a feature mining algorithm to select a feature subset of informative DNASDs, and acquires rule-based knowledge based on the selected feature subset. The mining algorithm using an optimization approach to feature selection identifies the most informative and discriminating DNASDs among human and *Drosophila* species. The top-ranked rules reveal that the global sequence descriptor, the length of nucleotide A at the first position of the sequence, and two physicochemical properties, absorption maxima and molecular weight, are efficient in distinguishing promoters from nonpromoters in human and *Drosophila* species, respectively. Additionally, this work analyzes the contributions of a feature set of DNA sequence descriptors to the promoter prediction using the MED values. The three physicochemical properties of absorption maxima, molecular weight, and molar absorption coefficient have high MED values, meaning the three properties are clearly useful in distinguishing promoters from nonpromoters in human and *Drosophila* species. Future work on PromHD will develop a well-characterized promoter system for synthetic biology applications. Moreover, we believe that this proposed method will also be effective in designing prediction methods for other DNA sequence-based applications. The promoter prediction system by using PromHD has been implemented at http://iclab.life.nctu.edu.tw/promhd. All used datasets were given in the website.

## Figures and Tables

**Table 1:** Conventional features for promoter prediction.

Features	Label
Context features	
*k*-mere frequency	A1
Transition	A2
Distribution	A3
Entropy density profile	A4
Codon-position-independent frequencies of mononucleotides	A5
Digitized DNA sequence	A6
Position-specific information	A7
Relative entropy	A8
Flanking genomic sequence	A9
Signal features	
TATA	B1
5′UTR (untranslated region)	B2
Exons region	B3
Intron region	B4
3′UTR	B5
Downstream promoter element	B6
TFIIB recognition element	B7
Motif ten element	B8
CCAAT	B9
GC	B10
Transcription factor binding site	C
CpG islands	D
Structural features	
DNA curvature	E1
DNA flexibility	E2
Stabilizing energy of Z-DNA	E3
DNA denaturation values	E4
Base stacking energy	E5
Nucleosome positioning preference	E6
Dinucleotide free energy	E7
Tri-nucleotide CG content	E8
DNA bendability	E9
DNA-bending stiffness	E10
A-philicity	E11
Protein induced deformability	E12
Propeller twist	E13
B-DNA twist	E14
Protein-DNA twist	E15
Duplex stability (disrupt energy)	E16
Duplex stability (free energy)	E17
Radical cleavage intensity	E18
Z-DNA	E19
Epigenetic features	F

**Table 2:** Some representative prediction methods and classifiers with their used features. The informative features are explained in Table 1.

Methods	Classifier	Features
ARTS [16]	SVM	B2, E5, E13
CorePromoter [20]	Stepwise strategy	B1, B6, C
CoreBoost [23]	LogitBoost algorithm with decision trees	A1, B1, B9, B10, C, D, E2
CoreBoost_HM [22]	Hidden Markov model	A1, B1, B9, B10, C, D, E2, F
CpGcluster [13]	Distance-based algorithm	D
CpGProD [14]	A generalized linear model	D
DragonGSF [12]	Artificial neural network	B9
DragonPF [15]	Artificial neural network	D
EP3 [28]	Analysis approach	E3–18
Eponine [34]	Relevance vector machine	B1
FSPP [41]	SVM	E4–6, E10–17
FirstEF [18]	Decision tree	B4, D
Fuzzy-AIRS [40]	Artificial immune recognition system	A1
GDZE [6]	Fisher's linear discriminant algorithm	A1–5, E7
GSD-FLD [6]	Fisher's linear discriminant algorithm	A1–4
HMM-SA [33]	Hidden Markov model, simulated annealing	F
McPromoter [51]	Artificial neural network, hidden Markov model	E3–6, E8–17
NNPP2.2 [37]	Artificial neural network	B1, B4
Nscan [52]	Hidden Markov model, Bayesian networks	B2–5
Prom-Machine [39]	SVM	A1 (128 top-ranked 4-mer motifs)
PromPredict [53]	A scoring function and threshold values	A10, B12, E1, E7, E9, E17
Promoter 2.0 [19]	Neural networks and genetic algorithms	B1, B4, B9, B10
PromoterExplorer [8]	AdaBoost algorithm	A1, A6, D
PromoterInspector [54]	Context analysis approach	A1
PromoterScan [55]	Linear discriminant analysis	B1, C
ProSOM [30]	Artificial neural network	E5, E7
PSPA [9]	Probabilistic model	A1, A7
TSSW [56]	Linear discriminant function	B1
vw Z-curve [7]	Partial least squares	A5
Wu method [10]	Linear discriminant analysis	A3–5, A7, A8

**Table 3:** Three physicochemical properties of nucleotide.

DNASD	Description	Nucleotide				Rank by MED	
		A	C	G	T	Human	DPL
*D*_AM_	Absorption maxima (determined at pH 7.0)	259	271	253	267	2	1
*D*_MW_	Molecular weight	491.2	467.2	507.2	482.2	7	2
*D*_MAC_	Molar absorption coefficient	15200	9300	13700	9600	11	3

**Table 4:** Top 20 descriptors of 4-mer motifs. Top 20 descriptors of the 4-mer motifs are contained in the reference set of 167 DNASDs. The descriptors of the TATA motif are ranked at the 199th and 98th when applied for the HPL and DPL datasets, respectively.

Rank	HPL dataset			DPL dataset		
	4-mer motif	Score	Included (*m* = 99)	4-mer motif	Score	Included (*m* = 74)
1	TGAA	1000	+	AAAG	1000	+
2	TGAT	941	+	AAGA	956	+
3	CCGG	878	−	TTCG	948	+
4	TATG	843	+	AGAA	922	−
5	TGGA	817	−	GAAA	866	−
6	GATG	770	+	AAGG	791	+
7	TCAA	739	+	CGCC	787	−
8	TACA	702	+	AGAT	777	−
9	AGGC	697	−	AATA	759	−
10	ATGA	694	+	TCGC	747	−
11	TTGA	672	+	TGAT	744	+
12	CGGC	662	−	TGAA	732	−
13	CAGG	651	−	ATCG	732	+
14	ATGT	634	−	TCGA	724	+
15	AGCG	633	−	CGGT	724	−
16	CGCG	629	−	ATAA	712	+
17	AGCC	618	−	CGAT	710	−
18	TCAT	595	−	CGCG	703	−
19	GAGC	592	+	GAAG	699	+
20	AGGG	582	−	ATAG	697	−
⋮	⋮	⋮	⋮	⋮	⋮	⋮
				25 AAGT	642	
⋮	⋮	⋮	⋮	⋮	⋮	⋮
199	TATA	111	−	98 TATA	365	−

+: included in the set of *m* DNASDs.

−: not included in the set of *m* DNASDs.

**Table 5:** Comparisons of training and test accuracies (ACC, %), sensitivity (SN), specificity (SP), and MCC for the HP dataset.

Method	No. of used features (*C*, *γ*)	10-CV HPL				Independent test HPT			
		ACC	SN	SP	MCC	ACC	SN	SP	MCC
SVM-GSD	36 (2^8^, 2^−2^)	97.4	0.972	0.976	0.949	93.6	0.930	0.941	0.872
SVM-4mer	128 (2^3^, 2^−3^)	94.2	0.949	0.936	0.885	91.0	0.953	0.867	0.823
SVM-RBS	82 (2^7^, 2^−5^)	96.0	0.964	0.956	0.920	91.9	0.885	0.962	0.840
PromHD	99 (2^7^, 2^−5^)	98.9	0.979	0.979	0.979	96.4	0.967	0.960	0.927

**Table 6:** Comparisons of training and test accuracies (ACC, %), sensitivity (SN), specificity (SP), and MCC for the DP dataset.

Method	No. of used features (*C*, *γ*)	10-CV DPL				Independent test DPT			
		ACC	SN	SP	MCC	ACC	SN	SP	MCC
SVM-GSD	36 (2^2^, 2)	95.1	0.956	0.946	0.902	89.2	0.789	0.996	0.802
SVM-4mer	128 (2^3^, 2^−6^)	96.4	0.960	0.967	0.952	94.6	0.912	0.981	0.830
SVM-RBS	31 (2^7^, 1)	95.3	0.959	0.946	0.906	80.5	0.612	0.996	0.660
PromHD	74 (2^4^, 1)	99.3	0.996	0.990	0.986	97.5	0.961	0.988	0.949

**Table 7:** The rule-based knowledge of promoter prediction in human and *Drosophila* species.

Species	Rule-based knowledge			CF	Rules	Accuracy
(Human)						
R1-p:	If *D*_*D*_ (A, 1st) > 0.0177542	Then	Promoter	0.928	1	50.0%
R2-n:	If *D*_*D*_ (A, 1st) ≤ 0.0177542	Then	Non-promoter	0.999	1-2	96.2%
R3-n:	If *D*_MW_ > 0.284657 and *D*_*D*_ (A, 1st) ≤ 0.0950018 and *D*_*D*_ (C, 100%) ≤ 0.929016	Then	Non-promoter	0.985	1–3	99.5%
R4-n:	If *D*_C4_ (GCTC) > 0.0634629 and *D*_MW_ > 0.284657 and *D*_*D*_ (A, 1st) ≤ 0.0950018	Then	Non-promoter	0.974		
(*Drosophila*)						
R1-p:	If *D*_MW_≤ 0.280113 and *D*_AM_ > 0.27604	Then	Promoter	0.997	1	50.0%
R2-n:	If *D*_AM_≤ 0.27604	Then	Non-promoter	0.999	1-2	84.7%
R3-n:	If *D*_MW_ > 0.280113	Then	Non-promoter	0.997		

**Table 8:** Distribution of the extracted DNASDs.

	HPL		DPL		Common	
PCP	3	3(*D*_P_)	3	3(*D*_P_)	3	3(*D*_P_)
GSDs	23	4(*D*_E_), 2(*D*_C1_), 3(*D*_*T*_), 14(*D*_*D*_)	22	2(*D*_E_), 2(*D*_C1_), 3(*D*_*T*_), 15(*D*_*D*_)	15	1(*D*_E_), 2(*D*_C1_), 1(*D*_*T*_), 11(*D*_*D*_)
Frequency descriptors of 4-mer motifs	73	73(*D*_C4_)	49	49(*D*_C4_)	14	14(*D*_C4_)

Total	99		74		32	

The abbreviations *D*_P_, *D*_C1_, *D*_C4_, *D*_E_, *D*_*D*_, and *D*_*T*_ represent the descriptors of physicochemical property (PCP) and the global sequence descriptors (GSDs) of 1-mer motif, 4-mer motif, EDP, distribution, and transition, respectively.
